# Identification of heart rate dynamics during treadmill and cycle ergometer exercise: the role of model zeros and dead time

**DOI:** 10.12688/f1000research.153397.2

**Published:** 2024-11-01

**Authors:** Kenneth J. Hunt, Hanjie Wang

**Affiliations:** 1 rehaLab - the Laboratory for Rehabilitation Engineering ,Institute for Human Centred Engineering HuCE School of Engineering and Computer Science, Bern University of Applied Sciences,, Biel/Bienne, 2501, Switzerland

**Keywords:** heart rate dynamics, system identification, treadmill exercise, cycle ergometer exercise

## Abstract

**Background:**

The response of heart rate to changes in exercise intensity is comprised of several dynamic modes with differing magnitudes and temporal characteristics. Investigations of empirical identification of dynamic models of heart rate showed that second-order models gave substantially and significantly better model fidelity compared to the first order case. In the present work, we aimed to reanalyse data from previous studies to more closely consider the effect of including a zero and a pure delay in the model.

**Methods:**

This is a retrospective analysis of 22 treadmill (TM) and 54 cycle ergometer (CE) data sets from a total of 38 healthy participants. A linear, time-invariant plant model structure with up to two poles, a zero and a dead time is considered. Empirical estimation of the free parameters was performed using least-squares optimisation. The primary outcome measure is model fit, which is a normalised root-mean-square model error.

**Results:**

A model comprising parallel connection of two first-order transfer functions, one with a dead time and one without, was found to give the highest fit (56.7 % for TM, 54.3 % for CE), whereby the non-delayed component appeared to merely capture initial transients in the data and the part with dead time likely represented the true dynamic response of heart rate to the excitation. In comparison, a simple first-order model without dead time gave substantially lower fit than the parallel model (50.2 % for TM, 47.9 % for CE).

**Conclusions:**

This preliminary analysis points to a linear first-order system with dead time as being an appropriate model for heart rate response to exercise using treadmill and cycle ergometer modalities. In order to avoid biased estimates, it is vitally important that, prior to parameter estimation and validation, careful attention is paid to data preprocessing in order to eliminate transients and trends.

## 1. Introduction

Heart rate is an easy-to-measure physiological variable that can be used to characterise the intensity of exercise, both quantitatively and qualitatively using categories such as light, moderate and vigorous. ^1^ The ability to regulate heart rate during exercise using feedback control would therefore allow accurate prescription of training regimes in both clinical and non-clinical settings. Since feedback controllers require a model of the dynamic response of heart rate during exercise, it is important to first consider the fidelity of different model structures.

It has been proposed that heart rate response to changes in exercise intensity comprises three main phases ^2^^,^^3^: an immediate, relatively small and fast Phase I; a slower, delayed and larger Phase II; and, if the exercise intensity exceeds the anaerobic threshold, a later and very slow Phase III drift.

This observation led to the investigation of empirical identification of dynamic models of heart rate using first- and second-order transfer functions for treadmill (TM) ^4^ and cycle ergometer (CE) ^5^ exercise. The second-order case was anticipated to capture Phase I and II response modes; but, since the models were intended to be used for analytical design of feedback controllers for heart rate, where integral action would cancel very slow drift, Phase III was not considered; in addition, to simplify feedback design, dead time was neglected.

Thus, in both of the preceding investigations of heart rate dynamics ^4^^,^^5^ the dynamic response of heart rate was modelled as nominal linear transfer functions $P_{o}\left(s\right)$ of first (P1) and second (P2) order:

$$P1:\;P_{o}\left(s\right)=\frac{k}{\mathrm{\tau s}+1},\;\;P2:\;P_{o}\left(s\right)=\frac{k}{\left(\tau_{1}s+1\right)\left(\tau_{2}s+1\right)}$$ (1)

where k is a steady-state gain and the τ ’s are time constants.

It was found that second-order models gave substantially and significantly better model fidelity compared to the first order case (TM, ^4^ CE ^5^) and that feedback control of heart rate was more accurate when based on second-order models (TM, ^6^ CE ^5^).

But the classical Phase I - Phase II model of heart rate response ^2^^,^^3^ comprises the parallel connection of two first-order models, i.e. the sum of a first-order transfer function of the form P1 above and a P1 with pure delay. Theoretically, this would lead to a second-order model with two poles, but also—when dead time is neglected—with a single zero. The effect of this (theoretical) zero was not reported in the previous studies ^4^^,^^5^ as it was found not to lead to any difference in empirical model fit, presumably due to overfitting. Furthermore, since the classical sources propose the addition of a dead time to one of the modes to capture the slightly later onset of the Phase II component, the inclusion of a pure delay warrants further attention.

The present work therefore aimed to perform a retrospective analysis of the previous investigations of heart rate dynamics during treadmill ^4^ and cycle ergometer ^5^ exercise to more closely consider the effect of including a zero and a dead time in the model. The respective datasets are available on the OLOS repository. ^7^^,^^8^

## 2. Methods

### 2.1 Data collection

Full details of experimental procedures employed for data collection in the preceding treadmill and cycle ergometer investigations can be found in the respective publications. ^4^^,^^5^ Essential elements of the protocols are summarised in this Brief Report.

For both exercise modalities, healthy, able-bodied participants exercised at moderate-to-vigorous intensity: in the treadmill analysis ^4^ there were 11 participants (8 male, 3 female; overall mean age 32.5 years, mean body mass 75.5 kg, mean height 1.79 m); for the cycle ergometer ^5^ there were 27 participants (20 male, 7 female; overall mean age 30.8 years, mean body mass 76.3 kg, mean height 1.79 m). Participants were required to be regular exercisers (30-min bouts, 3 times per week), non-smokers, and to be free of injury and illness.

A similar pseudo-random binary sequence (PRBS) input signal was employed in both cases to excite relevant modes of heart rate response dynamics. All participants performed two identical open-loop identification tests to facilitate counterbalanced cross-validation of model parameter estimates: consequently, there were 22 TM data sets and 54 CE data sets. All of these data sets were included in the present retrospective analysis.

**Figure 1.**
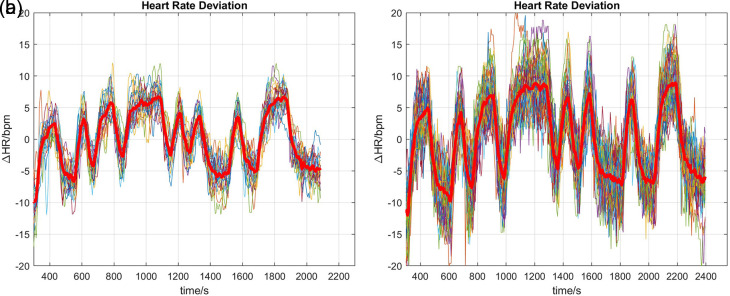
Heart rate measurements. In each plot, thin lines are the individual measurements (22 for TM, 54 for CE); the thick red lines are averages of the individual measurements. Data are plotted as deviations △ HR around mean heart rate levels. (a) Treadmill, (b) Cycle Ergometer.

To aid the following Discussion ( Sec. 4), all existing heart rate measurements that were included in the parameter estimation and validation procedures are illustrated ( Figure 1).

### 2.2 Model structures

In the present work, we consider a linear, time-invariant (LTI) plant model structure with up to two poles, a zero and a dead time, that maps an input signal *u* to the output *y*, namely

$$P_{o}\left(s\right)=\frac{k\left(T_{z}s+1\right)}{\left(\tau_{1}s+1\right)\left(\tau_{2}s+1\right)}e^{-T_{d}s}:\;u\mapsto y$$ (2)

where k is the steady-state gain, $\tau_{1}$ and $\tau_{2}$ are time constants (corresponding to real poles at $s=-1/\tau_{1}$ and $s=-1/\tau_{2}$ ), $T_{z}$ admits a zero $\left(s=-1/T_{z}\right)$ , and $T_{d}$ is a pure delay. The general model Eq. (2) can be constrained by choice of the τ ’s, $T_{z}$ and $T_{d}$ to several simpler structures as summarised in tabular form ( Table 1): in total, the seven model structures listed were considered in the present analysis; this includes one formed by the parallel connection of two first-order transfer functions, one with a dead time and one without, viz. P1∥P1D .

**Table 1.** Model structures.

Model	$P_{o}\left(s\right)$	constraints (cf. Eq. (2))
P1	$\frac{k}{\tau s+1}$	$T_{z}=0,T_{d}=0,\tau_{1}=\tau ,\tau_{2}=0$
P1D	$\frac{k}{\tau s+1}e^{-T_{d}s}$	$T_{z}=0,\tau_{1}=\tau ,\tau_{2}=0$
P2	$\frac{k}{\left(\tau_{1}s+1\right)\left(\tau_{2}s+1\right)}$	$T_{z}=0,T_{d}=0$
P2D	$\frac{k}{\left(\tau_{1}s+1\right)\left(\tau_{2}s+1\right)}e^{-T_{d}s}$	$T_{z}=0$
P2Z	$\frac{k\left(T_{z}s+1\right)}{\left(\tau_{1}s+1\right)\left(\tau_{2}s+1\right)}$	$T_{d}=0$
P2ZD	$\frac{k\left(T_{z}s+1\right)}{\left(\tau_{1}s+1\right)\left(\tau_{2}s+1\right)}e^{-T_{d}s}$	none
P1∥P1D	$\frac{k_{p1}}{\tau_{p1}s+1}+\frac{k_{p2}}{\tau_{p2}s+1}e^{-T_{d}s}$	N/A

Model names can be interpreted as follows: 1 = first order, 2 = second order, D = dead time, Z = zero.

P1∥P1D denotes parallel connection of P1 and P1D models.

N/A = not applicable.

The generic plant output signal y corresponds to heart rate [beats/min, bpm] while the input u depends on the exercise modality: for the treadmill, it is speed [m/s]; for the cycle ergometer, it is work rate [W]. As noted above, the input for both modalities took the form of a PRBS signal.

### 2.3 Parameter estimation and outcome measure

Empirical parameter estimation was performed using the Matlab System Identification Toolbox (The MathWorks, Inc., USA), wherefore, in the table ( Table 1), we have adopted model names corresponding to the terminology used in the toolbox. In general, models of the form Eq. (2) are referred to in the toolbox as “process models”.

Estimation of the free model parameters— k , the τ ’s, $T_{z}$ and $T_{d}$ in Eq. (2), constrained for the different model structures as indicated in Table 1—was done with the Matlab **procest** function using least-squares optimisation with regularly sampled time-domain data. ^9^ To focus the search algorithm, model parameters were constrained to lie in physiologically plausible ranges. As in our previous work ^4^^,^^5^ separate models were identified for each individual data set and counterbalanced cross-validation was employed by pairing the two measurements for each participant.

The primary outcome measure is model fit, which is a normalised root-mean-square model error (NRMSE):

$$\mathrm{fit}=\left(1-\sqrt{\frac{\sum_{i=1}^{N}\;\;{\left(y\left(i\right)-y_{\mathrm{sim}}\left(i\right)\right)}^{2}}{\sum_{i=1}^{N}\;\;{\left(y\left(i\right)-\bar{y}\right)}^{2}}}\right)$$ (3)

where $\bar{y}$ is the mean heart rate and $y_{\mathrm{sim}}$ is the heart rate that was simulated using the estimated models. The summations range over the evaluation period up to the number of discrete data points included, N . A sample period of 5 s was used. Model fit was computed using the Matlab **compare** function.

## 3. Results

Goodness-of-fit values for the seven model structures and two exercise modalities are summarised in Table 2; the estimated model parameters are also tabulated ( Table 3). Average maximal heart rate for the treadmill was 158.4 bpm; for the cycle ergometer it was 140.2 bpm (this is in line with our setting the mean target heart rate for the CE to be 20 bpm lower than for the TM in order to achieve a similar level of perceived exertion). ^10^

**Table 2.** Mean model fit (normalised RMSE, Eq. (3), [%]).

Modality	P1	P1D	P2	P2D	P2Z	P2ZD	P1∥P1D
TM	50.2	54.0	54.5	54.5	53.9	55.2	56.7
CE	47.9	51.9	51.0	52.1	50.4	52.8	54.3

Values are averages of individual fits for n=22 TM models and n=54 CE models.

RMSE = root-mean-square error, TM = treadmill, CE = cycle ergometer.

**Table 3.** Model parameters for treadmill (TM) and cycle ergometer (CE).

Model	Modality	k/(bpm/[u])	$\tau_{1}/s$	$\tau_{2}/s$	$T_{z}/s$	$T_{d}/s$
P1	TM	28.6	70.6	-	-	-
	CE	0.46	68.8	-	-	-
P1D	TM	25.0	47.7	-	-	13.1
	CE	0.40	45.9	-	-	13.8
P2	TM	24.7	18.6	37.8	-	-
	CE	0.39	19.6	37.7	-	-
P2D	TM	23.9	13.7	37.8	-	5.4
	CE	0.38	15.5	33.2	-	6.9
P2Z	TM	24.1	24.9	40.2	7.3	-
	CE	0.38	31.2	46.2	18.7	-
P2ZD	TM	23.7	33.2	50.6	38.4	11.1
	CE	0.39	33.5	59.4	50.0	12.5
P1∥(P1D) *	TM	7.0	141.5	-	-	-
	CE	0.09	180.7	-	-	-
(P1)∥P1D *	TM	20.2	34.3	-	-	17.9
	CE	0.35	37.9	-	-	17.1

Values are averages of individual parameters for n=22 TM models and n=54 CE models.

[u] - units of the input signal: for the TM, m/s; for the CE, W.

* For the P1∥P1D model structure, parameters are shown separately for the P1 (second-bottom row) and P1D (bottom row) components: k and $\tau_{1}$ correspond respectively to $k_{p1}$ and $\tau_{p1}$ , or $k_{p2}$ and $\tau_{p2}$ , in the bottom row of Table 1.

## 4. Discussion

Goodness-of-fit outcomes for the treadmill and cycle ergometer followed a similar pattern. There was a substantial improvement in fit for P1D vs. P1, indicating the clear presence of dead time in heart rate response; $T_{d}$ for P1D was similar for TM and CE at 13.1 s and 13.8 s, respectively ( Table 3).

Model fit for P2, P2D and P2Z was similar to P1D, while P2ZD showed a further slight improvement. It has to be remarked, however, that estimated $T_{z}$ values for individual models varied widely on the range -15 s to 100 s, thus displaying in part negative-phase behaviour (i.e. $T_{z}<0$ ). Furthermore, fit for P2Z was slightly lower than for P1D, P2 and P2D. Taken together, these observations point to a degree of overfitting when a plant zero is included.

Having excluded further consideration of models with a zero, we note a further substantial increase in fit for the parallel P1∥P1D model structure when compared to P1D, P2 and P2D. A critical observation in this regard is that the P1 parameters in the P1∥P1D structure displayed very small gains and very large time constants when compared to the parallel-models’ P1D parameters ( Table 3): for the TM, the gains were 7.0 bpm/(m/s) and 20.2 bpm/(m/s), (P1 vs. P1D), and the time constants 141.5 s vs. 34.3 s; for the CE, gains were 0.09 bpm/W vs. 0.35 bpm/W and time constants 180.7 s vs. 37.9 s.

A likely explanation for this apparent anomaly can be gleaned by perusal of the heart rate measurements ( Figure 1). It can be seen that there is a small yet clearly discernible drift in heart rate during the first few minutes of the responses, with the duration of drift in line with the observed P1 time constants 141.5 s (TM, Figure 1a) and 180.7 s (CE, Figure 1b). It is therefore plausible that the P1 part of the P1∥P1D model merely reflects the initial transient, while the P1D part represents the true dynamic response of heart rate to the excitation. Care should therefore be taken in future investigations to exclude initial transients and slow trends prior to parameter estimation and validation.

The gains and time constants are seen to be somewhat lower for the P1D part of the P1∥P1D model than for the P1D-only model (gains 20.2 bpm/(m/s) vs. 25.0 bpm/(m/s) for TM, 0.35 bpm/W vs. 0.40 bpm/W for CE; time constants 34.3 s vs. 47.7 s for TM, 37.9 s vs. 45.9 s for CE; Table 3), and the dead times somewhat higher (17.9 s vs. 13.1 s for TM, 17.1 s vs. 13.8 s for CE). These differences are likely due to model bias introduced in the P1D-only model as a consequence of the initial drift in heart rate, as discussed above.

As noted in previous reports ^4^^,^^5^ second-order models of the form P2 gave substantially and significantly better fidelity than first-order models P1 (cf. Table 2). However, the identification here of a substantial dead time, coupled with the observed superiority of the P1D part of the parallel P1∥P1D model (following elimination of heart rate drift), suggests that the second time constant in the P2 model may simply have partially absorbed the neglected time delay rather than having modelled any underlying dynamic mode in the heart rate response.

A final observation is that the time constants for the TM and CE, when compared for all seven model structures, are in strikingly close agreement ( Table 3). This is in line with a previous comparison of heart rate dynamics between the TM and CE modalities that showed no significant difference in the time constant of heart rate response. ^10^

Due to the retrospective nature of this investigation—that used existing data sets—the results and conclusions are considered to be provisional, but they do provide insights for the design of future studies: to avoid the confounding effect of initial transients, the plant input test signal should be designed to ensure that a physiological steady state has been reached in advance of the data evaluation period; a formal, statistical study design should be employed for comparison of the different model structures - the results of the present work provide effect-size estimates for statistical power and sample size calculations.

## 5. Conclusions

This preliminary analysis points to the P1D structure—that is to say, a linear first-order system with dead time—as being an appropriate model for heart rate response to exercise using treadmill and cycle ergometer modalities. In order to avoid biased estimates, it is vitally important that, prior to parameter estimation and validation, careful attention is paid to data preprocessing in order to eliminate transients and trends.

### Ethical considerations

The study that generated both the treadmill and cycle ergometer datasets was performed in accordance with the Declaration of Helsinki; the study was reviewed and approved by the Ethics Committee of the Swiss Canton of Bern (Ref. 2019-02184; approval date 16 January 2020). Participants provided written, informed consent prior to inclusion in the study.

## Authors’ contributions

Both authors made substantial contributions to the conception and design of the study; HW did the treadmill data acquisition; KH and HW performed the data analysis; both authors contributed to the interpretation of the data. KH drafted the manuscript; HW reviewed it critically for important intellectual content. Both authors read and approved the final manuscript.

## Data Availability

The datasets analysed in this research are available in the OLOS repository as follows: Identification of heart rate dynamics during treadmill exercise: comparison of first- and second-order models - Treadmill dataset, https://doi.org/10.34914/olos:bivq3dcebff5dfrqtbf3v5y7si. ^7^ Heart rate dynamics identification and control in cycle ergometer exercise: Comparison of first- and second-order performance --Cycle ergometer dataset, https://doi.org/10.34914/olos:xtyv7akiu5bzdba3oemrarg4ru. ^8^ Please click on the link, then click on the “Files” tab at the bottom right of the screen to access the data. Data is available under the terms of the Creative Commons Attribution 4.0 International license.

## Software

Data analysis was conducted using third-party proprietary software (Matlab, Release 2024a, The MathWorks, Inc., USA). There is no open-source alternative that performs the specific functions employed. The Methods section of this article provides sufficient information to allow replication of the analysis, i.e. Matlab function names and mathematical definitions of the outcome measures.
