# Supplementary material for: Identification of heart rate dynamics during treadmill and cycle ergometer exercise: the role of model zeros and dead time
Source: F1000Res. 2024 Nov 1;13:894. Originally published 2024 Aug 6. [Version 2] doi: 10.12688/f1000research.153397.2 (PMC11550391; doi:10.12688/f1000research.153397.2)
Supplement: Peer review history

# Reviewer report: Reviewer response for version 2

**DOI:** 10.5256/f1000research.173966.r336843

**Published:** 2024-11-09

**Authors:** Lauren Miutz

**Affiliations:** 1 Department of Health and Sport Science, University of Dayton, Dayton, Ohio, USA; 2 Faculty of Kinesiology, University of Calgary, Calgary, Canada

Appreciate the authors addressing my previous comments and adding the additional information to the article.

Is the work clearly and accurately presented and does it cite the current literature?

Yes

If applicable, is the statistical analysis and its interpretation appropriate?

Yes

Are all the source data underlying the results available to ensure full reproducibility?

Yes

Is the study design appropriate and is the work technically sound?

Yes

Are the conclusions drawn adequately supported by the results?

Yes

Are sufficient details of methods and analysis provided to allow replication by others?

Partly

Reviewer Expertise:

Exercise Physiology

I confirm that I have read this submission and believe that I have an appropriate level of expertise to confirm that it is of an acceptable scientific standard.

# Reviewer report: Reviewer response for version 2

**DOI:** 10.5256/f1000research.173966.r336844

**Published:** 2024-11-06

**Authors:** Przemysław Seweryn Kasiak

**Affiliations:** 1 Department of Internal Medicine and Cardiology, Medical University of Warsaw, Warsaw, Poland

I'm grateful for the Authors for applying necessary revision. 

 I also congratulate them on their article. Finally, please reconsider adding a proposed reference.

Is the work clearly and accurately presented and does it cite the current literature?

Partly

If applicable, is the statistical analysis and its interpretation appropriate?

Partly

Are all the source data underlying the results available to ensure full reproducibility?

Yes

Is the study design appropriate and is the work technically sound?

Yes

Are the conclusions drawn adequately supported by the results?

Partly

Are sufficient details of methods and analysis provided to allow replication by others?

No

Reviewer Expertise:

Sports Cardiology

I confirm that I have read this submission and believe that I have an appropriate level of expertise to confirm that it is of an acceptable scientific standard.

## References

[1] : Validity of the Maximal Heart Rate Prediction Models among Runners and Cyclists. *J Clin Med* .2023;12(8) : 10.3390/jcm12082884 10.3390/jcm12082884 37109218 PMC10146295

# Reviewer report: Reviewer response for version 1

**DOI:** 10.5256/f1000research.168288.r331353

**Published:** 2024-10-24

**Authors:** Lauren Miutz

**Affiliations:** 1 Department of Health and Sport Science, University of Dayton, Dayton, Ohio, USA; 2 Faculty of Kinesiology, University of Calgary, Calgary, Canada

Brief description of article: Reanalyzed retrospective data to better identify a model of best fit for heart rate changes during exercise. The present study included a zero point and a pure delay in the heart rate model.

 Relevance: Although the research reported that the model including dead time (compared to the model without dead time) provided a “better fit,” is the this clinically meaningful. The researchers reported a change in percentage of fit, however in the field of clinical or non-clinical exercise physiology, heart rate (especially regarding exercise) is largely provided as a range. Thus, are the percentages provided, between the two models, clinically relevant within the field.

 Major points:

 • Additional detail around the sample is needed, simply referencing the original work is not sufficient.

 • Information around resting average heart rate (cycle and treadmill) and average maximal heart rate (cycle and treadmill) should be provided.

 Minor point:

 • Training status of the participants should be reports as heart rate (and potentially the delay and amount of dead time) are often linked to one’s training status, especially aerobic capacity.

 • Interesting findings, however when reanalyzing retrospective data the researchers need to be careful the questions being answered is relevant and that adequate detail is provided to the reader, regardless of their knowledge surrounding the original study/data set.

Is the work clearly and accurately presented and does it cite the current literature?

Yes

If applicable, is the statistical analysis and its interpretation appropriate?

Yes

Are all the source data underlying the results available to ensure full reproducibility?

Yes

Is the study design appropriate and is the work technically sound?

Yes

Are the conclusions drawn adequately supported by the results?

Yes

Are sufficient details of methods and analysis provided to allow replication by others?

Partly

Reviewer Expertise:

Exercise physiology; cerebrovascular physiology; sport-related concussion & exertional measures

I confirm that I have read this submission and believe that I have an appropriate level of expertise to confirm that it is of an acceptable scientific standard, however I have significant reservations, as outlined above.

# Response

**Published:** 2024-10-24

**Authors:** Kenneth Hunt

**Affiliations:** Engineering & Computer Science, Berner Fachhochschule, Bern, Canton of Bern, Switzerland

Dear Reviewers

 Many thanks for your detailed and constructive comments on our manuscript. We are pleased to provide a point-by-point response below. Changes to the manuscript have been implemented as noted below and submitted using “track changes” in the revised manuscript.

 With best wishes.

 Yours faithfully

 The authors

**Reviewer #2 Report and Response**: Lauren Miutz

 Brief description of article: Reanalyzed retrospective data to better identify a model of best fit for heart rate changes during exercise. The present study included a zero point and a pure delay in the heart rate model.

**RESPONSE**: We would like to thank you for your careful reading of our article and for your constructive comments. Please find below our point-by-point response.

 Relevance: Although the research reported that the model including dead time (compared to the model without dead time) provided a “better fit,” is the this [sic] clinically meaningful. The researchers reported a change in percentage of fit, however in the field of clinical or non-clinical exercise physiology, heart rate (especially regarding exercise) is largely provided as a range. Thus, are the percentages provided, between the two models, clinically relevant within the field.

**RESPONSE**: Thank you for this remark regarding relevance for clinical or non-clinical exercise physiology. In a similar vein, Reviewer #1 requested more information regarding the clinical and physiological perspectives, and practical applications. To address these points, we added the following paragraph at the start of the Introduction:

 “Heart rate is an easy-to-measure physiological variable that can be used to characterise the intensity of exercise, both quantitatively and qualitatively using categories such as light, moderate and vigorous [10]. The ability to regulate heart rate during exercise using feedback control would therefore allow accurate prescription of training regimes in both clinical and non-clinical settings. Since feedback controllers require a model of the dynamic response of heart rate during exercise, it is important to first consider the fidelity of different model structures.”

 Furthermore, we would like to remark that, in the field of systems and control engineering—which is the field within which we authors work—model fidelity is usually provided either in absolute terms as a root-mean-square error (RMSE) or as a normalised RMSE, i.e. model fit, expressed in percent. We cannot definitively answer the question of whether the reported improvement in fit will be relevant to researchers or practitioners in other fields (e.g. exercise physiologists) and must leave it up to colleagues in those fields to assess our findings for themselves.

 Major points:

 • Additional detail around the sample is needed, simply referencing the original work is not sufficient.

**RESPONSE**: Thank you for this recommendation (a similar request was made by Reviewer #1). We do agree that it would be helpful to the reader to have more information in the current article. As noted in our response to Reviewer #1, we have added more details to the Methods, as follows:

 “For both exercise modalities, healthy, able-bodied participants exercised at moderate-to-vigorous intensity: in the treadmill analysis ^3^ there were 11 participants (8 male, 3 female; overall mean age 32.5 years, mean body mass 75.5 kg, mean height 1.79 m); for the cycle ergometer ^4^ there were 27 participants (20 male, 7 female; overall mean age 30.8 years, mean body mass 76.3 kg, mean height 1.79 m). Participants were required to be regular exercisers (30-min bouts, 3 times per week), non-smokers, and to be free of injury and illness.”

 • Information around resting average heart rate (cycle and treadmill) and average maximal heart rate (cycle and treadmill) should be provided.

**RESPONSE**: Thank you for this suggestion. We have added information on average maximal heart rate (cycle and treadmill) to the Results section, as quoted below. Unfortunately, we cannot provide average resting heart rate because recording was started at the onset of the PRBS input signal: resting heart rate was not recorded (resting HR played no role in the analysis).

 Text added to Results:

 “Average maximal heart rate for the treadmill was 158.4 bpm; for the cycle ergometer it was 140.2 bpm (this is in line with our setting the mean target heart rate for the CE to be 20 bpm lower than for the TM in order to achieve a similar level of perceived exertion [9]).”

 Minor point:

 • Training status of the participants should be reports [sic] as heart rate (and potentially the delay and amount of dead time) are often linked to one’s training status, especially aerobic capacity.

**RESPONSE**: Thank you for this comment. As noted above, we have now provided information regarding participants’ training status: “Participants were required to be regular exercisers (30-min bouts, 3 times per week) …”. Beyond this requirement (it was a formal inclusion criterion), we did not record any further information in this regard.

 • Interesting findings, however when reanalyzing retrospective data the researchers need to be careful the questions being answered is relevant and that adequate detail is provided to the reader, regardless of their knowledge surrounding the original study/data set.

**RESPONSE**: Many thanks for your observations, which are valuable. We hope that our responses and the corresponding revision of the manuscript now provide adequate detail independent of the original publications.

# Reviewer report: Reviewer response for version 1

**DOI:** 10.5256/f1000research.168288.r317159

**Published:** 2024-08-27

**Authors:** Przemysław Seweryn Kasiak

**Affiliations:** 1 Department of Internal Medicine and Cardiology, Medical University of Warsaw, Warsaw, Poland

Firstly, I would like to thank for inviting me as a Referee of this study. This research touches an important topic of heart rate during exercise and provide useful piece of knowledge for clinicians and sport practitioners. I am providing my comments for the Authors below.

 Major issues:

- I suggest more precise description of the rationale for this study from clinical and physiological perspective. Perhaps, an additional paragraph in the introduction would be welcome.
- Similarly, there is a lack of information about practical applications of this study in the discussion.

Minor issues:

- I suggest providing a brief description of the study population in the abstract.
- If the Authors will meet the word limit, I strongly recommend enhancing the information about study group (their demographics, fitness level etc.) and testing protocols.  
- Did the Authors evaluate sample size to ensure credibility of their analysis and conclusions? There are only 38 participants in this study.

In summary, this study is interesting. Further analysis of the preliminary report would be helpful for deep understanding of heart rate response to exercises.

Is the work clearly and accurately presented and does it cite the current literature?

Partly

If applicable, is the statistical analysis and its interpretation appropriate?

Partly

Are all the source data underlying the results available to ensure full reproducibility?

Yes

Is the study design appropriate and is the work technically sound?

Yes

Are the conclusions drawn adequately supported by the results?

Partly

Are sufficient details of methods and analysis provided to allow replication by others?

No

Reviewer Expertise:

Sports Cardiology

I confirm that I have read this submission and believe that I have an appropriate level of expertise to confirm that it is of an acceptable scientific standard, however I have significant reservations, as outlined above.

# Response

**Published:** 2024-10-24

**Authors:** Kenneth Hunt

**Affiliations:** Engineering & Computer Science, Berner Fachhochschule, Bern, Canton of Bern, Switzerland

Dear Reviewers

 Many thanks for your detailed and constructive comments on our manuscript. We are pleased to provide a point-by-point response below. Changes to the manuscript have been implemented as noted below and submitted using “track changes” in the revised manuscript.

 With best wishes.

 Yours faithfully

 The authors

**Reviewer #1 Report and Response**: Przemysław Seweryn Kasiak

 Firstly, I would like to thank for inviting me as a Referee of this study. This research touches an important topic of heart rate during exercise and provide useful piece of knowledge for clinicians and sport practitioners. I am providing my comments for the Authors below.

**RESPONSE**: Dziękuję bardzo—and thank you very much for your helpful comments, especially for your observation of relevance for clinicians and sport practitioners.

 Major issues:

- I suggest more precise description of the rationale for this study from clinical and physiological perspective. Perhaps, an additional paragraph in the introduction would be welcome.
- Similarly, there is a lack of information about practical applications of this study in the discussion.

**RESPONSE**: Thank you for these suggestions. We agree it is very useful to provide more information regarding the clinical and physiological perspectives, and practical applications. To address both of these points, we have added the following paragraph at the start of the Introduction:

 “Heart rate is an easy-to-measure physiological variable that can be used to characterise the intensity of exercise, both quantitatively and qualitatively using categories such as light, moderate and vigorous [10]. The ability to regulate heart rate during exercise using feedback control would therefore allow accurate prescription of training regimes in both clinical and non-clinical settings. Since feedback controllers require a model of the dynamic response of heart rate during exercise, it is important to first consider the fidelity of different model structures.”

 New reference [10]:

 10. Riebe D, Ehrman JK, Liguori G, Magal M, editors. ACSM’s guidelines for exercise testing and prescription. 10th ed. Philadelphia: Wolters Kluwer; 2018.

 Minor issues:

- I suggest providing a brief description of the study population in the abstract.

**RESPONSE**: Regrettably, the Abstract already has 298 words, with a limit of 300 words. But we have provided more detailed information on the study population in the main text as noted below.

  

- If the Authors will meet the word limit, I strongly recommend enhancing the information about study group (their demographics, fitness level etc.) and testing protocols.

**RESPONSE**: Thank you for this suggestion. As mentioned in the text, this is a retrospective analysis of data from two previously-published studies. We pointed out that full information regarding the study groups and testing protocols can be found in the corresponding citations, viz. references [3] and [4], as follows:

 “Full details of experimental procedures employed for data collection in the preceding treadmill and cycle ergometer investigations can be found in the respective publications. ^3^^ ,^^4^ Essential elements of the protocols are summarised in this Brief Report.”

 Nevertheless, we do agree it would be helpful to the reader to have more information in the current article, for which reason we have added more details to the Methods, as follows:

 “For both exercise modalities, healthy, able-bodied participants exercised at moderate-to-vigorous intensity: in the treadmill analysis ^3^ there were 11 participants (8 male, 3 female; overall mean age 32.5 years, mean body mass 75.5 kg, mean height 1.79 m); for the cycle ergometer ^4^ there were 27 participants (20 male, 7 female; overall mean age 30.8 years, mean body mass 76.3 kg, mean height 1.79 m). Participants were required to be regular exercisers (30-min bouts, 3 times per week), non-smokers, and to be free of injury and illness.”

  

- Did the Authors evaluate sample size to ensure credibility of their analysis and conclusions? There are only 38 participants in this study.

**RESPONSE**: Thank you for this important query. As noted above, the 38 participants came from two previous studies: a pilot treadmill study, [3], with 11 participants, and a cycle ergometer study, [4], with 27 participants.

 For the treadmill study, the sample size of n = 11 was chosen in accordance with the requirements of pilot studies, and no formal statistical power analysis was conducted.

 For the cycle ergometer study, an *a priori* statistical power and sample size estimate was included in the study protocol that was approved by our ethics committee. The details can be found in reference [4], as follows: “The sample size of n = 27 participants was estimated *a priori* by a statistical power calculation that used estimates of expected effect sizes and sample standard deviations obtained from previous studies in this lab, with the significance level of 5 % and a statistical power of 80 % (1 − β = 0.8).”

 In both cases, no post-hoc statistical power calculation was performed because observed effect sizes and their uncertainty bounds supersede any *a priori* estimates, and because “Post-hoc power estimates … have been shown to be logically invalid and practically misleading”; quotation from:

 Dziak JJ, Dierker LC, Abar B. The Interpretation of Statistical Power after the Data have been Gathered. Curr Psychol. 2020 Jun;39(3):870-877.

https://doi.org/10.1007/s12144-018-0018-1
